# When technology precedes regulation: the challenges and opportunities of e-pharmacy in low-income and middle-income countries

**DOI:** 10.1136/bmjgh-2021-005405

**Published:** 2021-05-20

**Authors:** Rosalind Miller, Francis Wafula, Chima A Onoka, Prasanna Saligram, Anita Musiega, Dosila Ogira, Ikedichi Okpani, Ufuoma Ejughemre, Shrutika Murthy, Surekha Garimella, Marie Sanderson, Stefanie Ettelt, Pauline Allen, Devaki Nambiar, Abdul Salam, Emmanuel Kweyu, Kara Hanson, Catherine Goodman

**Affiliations:** 1 Department of Global Health and Development, London School of Hygiene and Tropical Medicine, London, UK; 2 Institute of Healthcare Management, Strathmore University, Strathmore Business School, Nairobi, Kenya; 3 Department of Community Medicine, University of Nigeria, Nsukka, Nigeria; 4 The George Institute for Global Health India, New Delhi, India; 5 National Primary Healthcare Development Agency, Abuja, Nigeria; 6 Delta State Government, Asaba, Nigeria; 7 Department of Health Services Research and Policy, London School of Hygiene and Tropical Medicine, London, UK; 8 @iLabAfrica, Strathmore University, Nairobi, Kenya

**Keywords:** health systems, public health, qualitative study, health policy

## Abstract

The recent growth of medicine sales online represents a major disruption to pharmacy markets, with COVID-19 encouraging this trend further. While e-pharmacy businesses were initially the preserve of high-income countries, in the past decade they have been growing rapidly in low-income and middle-income countries (LMICs). Public health concerns associated with e-pharmacy include the sale of prescription-only medicines without a prescription and the sale of substandard and falsified medicines. There are also non-health-related risks such as consumer fraud and lack of data privacy. However, e-pharmacy may also have the potential to improve access to medicines. Drawing on existing literature and a set of key informant interviews in Kenya, Nigeria and India, we examine the e-pharmacy regulatory systems in LMICs. None of the study countries had yet enacted a regulatory framework specific to e-pharmacy. Key regulatory challenges included the lack of consensus on regulatory models, lack of regulatory capacity, regulating sales across borders and risks of over-regulation. However, e-pharmacy also presents opportunities to enhance medicine regulation—through consolidation in the sector, and the traceability and transparency that online records offer. The regulatory process needs to be adapted to keep pace with this dynamic landscape and exploit these possibilities. This will require exploration of a range of innovative regulatory options, collaboration with larger, more compliant businesses, and engagement with global regulatory bodies. A key first step must be ensuring that national regulators are equipped with the necessary awareness and technical expertise to actively oversee this e-pharmacy activity.

**Summary box**

- The e-pharmacy sector has been rapidly growing in low-income and middle-income countries over the past decade, with the COVID-19 pandemic encouraging a further surge in online sales, and an associated rise in cybercrime.
- Online medicine sales are linked to both public health concerns, such as sale of prescription-only medicines without a prescription, and sale of substandard and falsified medicines; and cyber-security concerns, including consumer fraud and lack of data privacy.
- E-pharmacy may also present opportunities for enhancing access to medicines, particularly for those requiring regular medication for chronic conditions, or with problems accessing traditional pharmacy services.
- Regulation of the sector has not kept pace with these rapidly evolving, dynamic markets which operate with ease across national boundaries, and present distinct regulatory challenges.
- Regulators need to pay greater attention to this sector, ensure they have the technical expertise to supervise it and adapt regulatory process to take advantage of the opportunities e-pharmacy provides for enhancing traceability and transparency of medicine sales.

## Introduction

The sale of medicines over the internet represents a major disruption to pharmacy markets across the globe. E-pharmacy has proliferated since the first online sales to consumers in the late 1990s. The global e-pharmacy market is currently worth around US$81.6 billion, and expected to grow to US$244 billion by 2027.^1^ While initially the preserve of high-income countries (HICs), in the past decade it has been growing rapidly in low-income and middle-income country (LMIC) settings^2 3^; for example India’s share of the global market is US$9.3 billion with a compound annual growth rate of 18%.^4^

Lockdowns and self-isolation due to COVID-19 have further catalysed online medicine sales. Key market players in India have reported sales surges of 100%–200%.^5^ There have also been reports of a shift to more elderly customers with chronic conditions, and these new trends are expected to continue, at least to some degree, even after the pandemic subsides.

The rapid expansion of the e-pharmacy market has been largely uncontrolled, and accompanied by significant public health concerns, such as sale of prescription-only medicines (POMs) without a prescription, and inadequate information provision to patients.^6–8^ In addition, non-health-related risks include consumer fraud and lack of data privacy (ibid). However, e-pharmacy also presents potential opportunities for enhancing access to medicines. Purchasing medicines online can be quick, simple, convenient and private, and, as internet and smart phone penetration increases, e-pharmacy has the potential to improve access for the disabled, the elderly, and those living in rural areas.^9^

The regulatory environment within which e-pharmacy operates will be crucial in managing how these risks and opportunities play out. In this paper we explore e-pharmacy regulation in LMICs, drawing on a set of 18 key informant interviews we conducted in Kenya, India and Nigeria, as well as relevant literature. The interviews took place between September and December 2018 with the chief executive officers of e-pharmacies, health policymakers and senior regulatory officials, and were analysed thematically. We begin by describing the e-pharmacy business models, key risks to consumers and current regulatory responses in the above three countries, before turning to the challenges and opportunities these pose for effective regulation. We conclude by proposing next steps for policy and research.

## E-pharmacy business models

Interviewees reported that e-pharmacies can be provider-facing (business to business selling) or consumer-facing (business to consumer selling). They are predominately for-profit, with a few social enterprises. Most are consumer-facing, with two main business models: establishments that stock medicines and supply them via e-orders (inventory model), and those that serve as a link between consumers and existing physical pharmacies, often through a mobile phone application (marketplace model). The inventory model can be further divided into businesses that operate purely online, and hybrid models which operate both brick-and-mortar and online pharmacies. In response to COVID-19, many brick-and-mortar pharmacy chains have developed online platforms, general online retailers are increasingly stocking medicines, and new providers are also entering the market.

## Risks to consumers

### Dangers from inappropriate medicine provision

The main risks to consumers identified in the literature relate to the sale of medicines, particularly POMs, which can easily be ordered without a prescription from many e-pharmacies.^10 11^ This includes antibiotics, which if used incorrectly can contribute to antimicrobial resistance, declared a ‘global health security emergency’ by WHO.^12^ Another key concern is narcotic sales, which are associated with controlled prescription drug (CPD) use disorders. Well described in HICs,^13^ these are a growing phenomenon in LMICs; non-medicinal use of prescription opioids, over-the-counter cough syrups containing opioids and amphetamines have all been reported across Africa and Asia.^14^ CPD use disorders can lead to devastating effects, including cardiovascular complications, deadly overdose and severe mental ill-health (ibid). Further, e-pharmacy is argued to have fuelled the sale of falsified medicines,^15^ reflecting the trade’s anonymity and global reach. When the quantity of active ingredient falls outside the therapeutic range, falsified medicines can lead to treatment failure and even death. Additionally, the inclusion of harmful ingredients such as boric acid, toxic paint, and antifreeze can be fatal.^16^

In some cases, e-pharmacies employ doctors to write prescriptions, though our key informants reported that this was often without taking any patient history or even personal details. There is also disquiet relating to the storage, handling and delivery of medicines that require an unbroken cold chain. At a broader level, there is said to be a general lack of clinical oversight within many e-pharmacies.

### Data security and cybercrime

Data security and patient confidentiality are important concerns. Many sites do not secure customers’ information, post-purchase emails often contain unencrypted links to customer information and online transactions are inadequately protected.^17^ Such site vulnerabilities can lead to consumer fraud and inadequate data protection. Moreover, cybercrime related to the COVID-19 pandemic is reported to be extremely dynamic and growing, including online sales of bogus cures, counterfeit test kits and non-delivery scams.^18 19^

## The regulatory response

Existing regulatory systems and agencies in Kenya, Nigeria and India have been slow to adapt to e-pharmacy. Although key informants in all three countries viewed effective regulation as a priority, at the time of writing, none had enacted a specific regulatory framework. Better regulation is hindered by confusion over regulatory responsibilities, particularly reflecting where e-pharmacy falls between the jurisdictions of health and e-commerce.

The countries are at different stages in working towards e-pharmacy regulation (table 1). The Kenyan retail pharmaceutical sector is regulated by the Pharmacy and Poisons Act (Cap 244 of Kenyan Laws), which pre-dates Independence and is viewed as outdated, despite numerous amendments over the years.^20^ At present, there are no statutory provisions that directly govern e-pharmacy. The drug regulatory authority, the Pharmacy and Poisons Board, was navigating this gap by using an informal system whereby e-pharmacies sought a ‘letter of no objection’ to operate and has recently introduced a basic e-pharmacy registration process. In Nigeria, the body responsible for licensing pharmacies, the Pharmacy Council of Nigeria, is said to have developed rules to guide the regulatory process for e-pharmacy in 2016, but they have not yet been made public.

**Table 1:** Overview of regulation of brick-and-mortar and e-pharmacies in India, Kenya and Nigeria

	Relevant regulatory agencies; roles and responsibilities	Regulation of brick-and-mortar pharmacies	Regulation of e-pharmacies
India	Central Drugs Standard Control Organisation State Drug Regulatory Authorities Pharmacy Council of India	Pharmacy Act, 1948 Drugs and Cosmetic Act, 1940 The Drugs and Cosmetic Rules, 1945 The Pharmacy Practice Regulations, 2015 Drugs and Magic Remedies Act, 1954 (advertising)	Drugs and Cosmetic (Amendment) Rules, 2018 (pending) Federation of Indian Chambers of Commerce and Industry, self-regulation through voluntary code of conduct, 2016
Kenya	Pharmacy and Poisons Board (PPB)	Pharmacy and Poisons Act (Cap 244 of Kenyan Laws)	Informal letter of no objection from PPB; basic registration process recently introduced
Nigeria	Pharmacists Council of Nigeria National Agency for Food and Drug Administration and Control	Pharmacists Council of Nigeria Decree 1992 (Decree No. 91) Poison and Pharmacy Act (Cap 535), 1946 and 1947 National Agency for Food and Drug Administration and Control Decree 1993 (Decree No. 15) and Amendment Decree 1999 (Decree No. 19) Drugs and Unwholesome Processed Food Decree 1999 (Decree No. 25) Pharmacists Council of Nigeria (Disciplinary Tribunal Rules) 2000	Unpublished regulations for e-pharmacy since 2016

In India, medicine sales fall under the Drugs and Cosmetics Act (1940), the Drugs and Cosmetic Rules (1945) and the Pharmacy Act (1948).^21^ In 2015 the Drug Controller of India alerted e-pharmacies that they must comply with this legislation, yet the following year acknowledged that it was inadequate and incompatible with e-pharmacy. In 2016, the Federation of Indian Chambers of Commerce and Industry introduced a ‘self-regulation code of conduct’ for e-pharmacy.^22^ Later that year, a subcommittee of the Drugs Consultative Committee examined the issue, leading to amendments to the 1945 Rules which were released in draft form in August 2018, but not notified. The Draft Rules require e-pharmacies to register through a Central Licensing Authority, and include details of medicines excluded from online sales, for example, narcotics; inspection procedures (bi-annual carried out by the Central Licensing Authority); advertising constraints (no direct-to-consumer advertising); and record keeping requirements. These Rules have been pending approval for over 2 years.

Within healthcare more broadly, there is legislation governing personal health records, such as the National Health Act, 2014, in Nigeria,^23^ and the Electronic Health Records Standards in India,^24^ but nothing specifically aimed at online medicine sales. In Kenya, the Health Act 2017 allows for the establishment of a body to regulate health products and technologies.^25^ There is potential for this to cover e-pharmacies, but it has not yet been used. Regulation of information technology and e-commerce is also evolving rapidly, further stimulated recently by increased use of digital technologies during the COVID-19 pandemic. Examples of potentially relevant legislation include India’s Information Technology Act, 2000, and proposed Personal Data Protection Bill, and Kenya’s Information and Communications Act 2013. In Nigeria, the National Information Technology Development Agency (NITDA) Act (2007) through section 6 (c) provides for NITDA to develop guidelines for electronic governance and to monitor the use of electronic data interchange, where the use of electronic communication will improve the exchange of information.^26^

The discrepancy between the strict regulations (on paper) for brick-and-mortar pharmacies and the absence of regulation for e-pharmacies has been the source of considerable grievance for brick-and-mortar pharmacy owners who feel this does not represent a level playing field, and also have a vested interest in preventing the growth of e-pharmacy. This power struggle is most evident in India, where the Tamil Nadu Chemist and Druggist Association has (successfully) taken legal action against e-pharmacy firms, and the All India Association of Chemists and Druggists has organised nationwide strikes in protest at e-pharmacy operation.^27^ In July 2020, legal objections from almost 850 000 brick-and-mortar pharmacies were successful in overturning the inclusion of an e-pharmacy portal in the government’s COVID-19 contact-tracing app.^28^

## Regulatory challenges

We present four key challenges to regulating e-pharmacy, related to lack of consensus on regulatory models, capacity, regulating cross border trade, and risks of over-regulation.

### Lack of consensus on regulatory models

Lower-income countries might consider looking to the regulatory experience of their higher income counterparts, which have been grappling with e-pharmacy for longer.^29^ However, there is no consensus in upper-middle and HICs on appropriate regulations. For example, in Thailand it is illegal to sell medicines online; whereas in the UK and Germany e-pharmacies are regulated as an extension of physical pharmacies and incorporated into existing regulatory frameworks. While the UK has a relatively permissive environment, Germany is more restrictive, only allowing the e-pharmacy sales from a few select countries.

Arguably one of the largest challenges facing HICs is the plethora of illegal or rogue e-pharmacies that operate without licensure, offer POMs without a prescription, and sell counterfeit medicines and goods. A small number of criminal gangs own thousands of ever-renewing bogus pharmacy websites.^30^ To address this challenge, several HICs have placed emphasis on verification systems to identify legitimate e-pharmacies, through the ‘EU common logo’, US Digital Pharmacy Accreditation and Canada’s Canadian International Pharmacy Association (CIPA) certification mark. To receive accreditation US e-pharmacies must apply for a ‘.pharmacy’ domain, a simple way to signal quality to customers through the web address. Further, search engines such as Google, Yahoo! and Bing now require a ‘.pharmacy’ domain in order to use their advertising services. Despite these developments, most HIC governments would admit to facing major challenges in regulatory compliance.

### Regulatory capacity and governance

While there are well-established legal frameworks for brick-and mortar pharmacies in LMICs (table 1), in practice they are often staffed by unqualified personnel, over-the-counter sale of POMs is rampant, history taking and information provision is inadequate and clinically inappropriate medicines are provided, including misuse of antibiotics.^31–33^ More generally, governance in the pharmacy sector has been identified as poor, with high levels of corruption treated as normal.^34–36^ Regulatory agencies tasked with addressing these problems are under-staffed and overburdened. Against this backdrop, the added burden of regulating e-pharmacy is daunting. Traditional pharmaceutical regulators lack the skills to monitor online transactions and their complex links with shipping, online advertising and payment services; and lack the power and resources to control large companies. Substantial capacity building and resource commitment are identified as necessary but are not yet forthcoming.

### Regulating across borders

Added to these problems is the challenge of using national regulatory frameworks to control a market that operates with ease across geographical boundaries. In the study countries, restrictions on imports for personal use vary. In India, a doctor’s prescription should accompany personal shipments; in Kenya, import of up to 3 months’ supply of prescription drugs is permitted subject to approval by the Health Ministry; in Nigeria, import of medicines requires a license and is also limited to 3 months’ supply.^37^ In practice, these restrictions are rarely enforced, with customers easily purchasing from other countries. Regulators have no jurisdiction over the activities of e-pharmacies outside their national borders, which may operate under different systems for medicine approvals, marketing procedures and retail pharmacy regulation. These challenges are compounded by the reach and anonymity of e-pharmacies, and the ease of creating new websites and removing old ones.^38 39^

Despite these challenges, there is no global regulatory agency for e-pharmacy, and only limited international cooperation. INTERPOL coordinates an annual effort entitled ‘Operation Pangea’ to disrupt the sale of falsified medicines online, seizing 105 million units of medicine and making over 3000 arrests since 2008, but most seizures have been in HICs.^40^ Some independent organisations have set up international verification systems eg LegitScript, yet again LMIC coverage remains very limited.

### Risks of over-regulation

While current regulation is clearly inadequate, there was concern among our interviewees that the potential benefits of e-pharmacy could be stifled if future regulations were too stringent. Brushwood warns that tough regulation directed towards illegitimate e-pharmacies runs the risk of stifling innovation, while doing little to prevent inappropriate medicine use.^41^ He likens this response to the ‘war on drugs’ that law enforcement agencies in the USA have waged, with limited success; an inadvertent effect of trying to protect the public from narcotic drug addiction is many patients experiencing uncontrolled pain due to limited access to appropriate analgesia.

## Regulatory opportunities

While e-pharmacy presents many regulatory challenges, some aspects of its operation may facilitate regulatory control. We explore the possibilities of consolidation and improved traceability/transparency.

### Market consolidation

Currently, pharmacy markets in all three study countries are highly fragmented, characterised by a very high number of independent brick-and-mortar retailers and wholesalers. The growing e-pharmacy segment offers the potential for some degree of consolidation within these markets, leading to upstream economies of scale in website platforms, procurement and distribution. For example, in India, despite numerous start-ups, the online market remains dominated by a handful of players including 1mg, MedLife, Netmeds, Pharmeasy, Myra and CareOnGO. Such market concentration could improve the regulatory situation. First, larger companies may be less costly to regulate than the many small individual brick-and-mortar pharmacies, as they are easier to identify and much of the regulatory activity can take place through a central headquarters. Second, systems of internal quality control and self-regulation may be more likely in order to preserve brand identity. For example, some e-pharmacies in Kenya have instituted their own rules and safety checks, such as mechanisms to ensure POM sales are based on a valid prescription. A social enterprise reportedly only works with registered pharmacies that employ a registered pharmacist and sends them reminders when licenses need to be renewed. Such consolidation may also enhance control over procurement, potentially improving medicine quality. Finally, economies of scale and greater transparency of price comparisons^42^ could reduce prices, as it has in other sectors^43^; many e-pharmacies certainly claim to offer cheaper medicines than brick-and-mortar pharmacies,^44^ although there is currently little research evidence to confirm this.

### Traceability and transparency

In all three countries, the potential for e-pharmacy to improve the traceability and transparency of medicines sales was emphasised by interviewees. The online nature of e-pharmacy transactions is believed to allow for greater transparency, if relevant records are made available to regulators.^42^ A move towards e-prescriptions could enable prescription analysis, whereby regulators could verify authenticity of prescriptions and gather information regarding appropriateness of medicine use. Further, regulators could assess compliance with some regulations using online mystery shoppers, saving time and financial resources. The traceability of transactions online would allow for identification of details such as batch number and expiry dates, which in turn could enable the possibility of tracking counterfeit medicines and allow for quicker recall of medicines in the event of adverse drug reactions. The use of blockchain solutions may enable such possibilities, providing each medicine with a unique identification code that can be tracked at every stage of the distribution chain from manufacture to end-user, using tamper-proof encryption technologies.

## Next steps: an agenda for e-pharmacy policy and research

There is an opportunity for LMICs to use regulation to shape the e-pharmacy sector while it is still emerging, and, if appropriate, even encourage its expansion. This opportunity may be amplified by the COVID-19 pandemic, with health at the forefront of public and political consciousness, and online medicine sales (and associated scams) soaring. In fact we could be presented with a ‘window of opportunity’ for policy change, with the identification of the problem, the development of strategies to address it and the political appetite to do so beginning to converge.^45^ Advances in digital health governance, such as the Indian National Digital Health Mission and Telemedicine Practice Guidelines released in the context of COVID-19 in 2020, present an opportunity for principles and processes relevant for e-pharmacy regulation to be better articulated.^46 47^

Given the dynamism of e-pharmacy markets, countries should move quickly to address the lack of regulatory frameworks. A first step could be for regulators to open a dialogue with larger, more compliant businesses, and work together to develop best practice guidelines and regulatory structures. However, care must be taken to guard against regulatory capture and ensure that the fundamental principles of pharmacy practice are not compromised. Policy options include tightening statutory regulation (eg, enhanced licencing, website removal), allowing greater flexibility to improve the feasibility of compliance (eg, greater prescribing role for pharmacists) and improving monitoring (eg, blockchain software to track online transactions). Regulation may also be enhanced by introducing risk-based approaches, whereby resources are focused on those more likely to violate the rules.^48^ Statutory regulation may be combined with more persuasive approaches, such as codes of practice, consumer education, accreditation seals, restricted domains such as ‘.pharmacy’ and working with national domain name registries and other intermediary businesses such as credit card companies to restrict rogue websites. E-pharmacy could potentially catalyse new regulatory thinking across the entire retail pharmacy sector, including brick-and-mortar pharmacies.

Achieving these changes requires raising awareness among regulators about which firms are operating nationally; the key foreign players used by national residents; and common regulatory infringements. Further, regulators need appropriate technical expertise, requiring both training of existing staff and recruitment of information technology and e-commerce specialists. Regulating across borders will require a coordinated, global response, including enhanced international surveillance. Organisations working in this domain should look to expand their initiatives further among LMICs and share expertise with national regulators. Finally, inter-country learning through the exchange of lessons and best practices should be encouraged.

Research on e-pharmacy to support these developments remains limited. Moving forward, we propose a research agenda that (1) addresses the performance of e-pharmacies, in terms of quality of service provision, prices and equity of access; (2) considers how the interests and power of stakeholders in this market influence regulatory systems development and enforcement; (3) tracks the regulatory response and evaluates its effectiveness; and (4) supports the development of viable technologies to support the enforcement of regulatory frameworks.

## Conclusion

E-pharmacy is rapidly growing in LMICs and this trend is likely to continue, amplified by COVID-19 and expanding e-commerce ecosystems. Under-regulated e-pharmacy markets pose serious threats to public health through misuse of medicines; yet the opportunities e-pharmacy provides to increase access and quality should not be overlooked. Current regulations have not kept pace with technological innovation and HICs are yet to develop effective models for LMICs to draw on, with the latter still grappling with endemic regulatory infringement in brick-and-mortar pharmacies. It will be important for rigorous research to accompany the growth of this industry and the regulatory response as it evolves.

## Data Availability

Data are available upon request.
